# Gene Conversion amongst *Alu* SINE Elements

**DOI:** 10.3390/genes12060905

**Published:** 2021-06-11

**Authors:** Liliya Doronina, Olga Reising, Jürgen Schmitz

**Affiliations:** 1 Institute of Experimental Pathology, ZMBE, University of Münster, 48149 Münster, Germany; o_reis01@uni-muenster.de; 2 EvoPAD-RTG, University of Münster, 48149 Münster, Germany

**Keywords:** gene conversion, partial gene conversion, transposable elements, *Alu* subfamily conversion, homoplasy

## Abstract

The process of non-allelic gene conversion acts on homologous sequences during recombination, replacing parts of one with the other to make them uniform. Such concerted evolution is best described as paralogous ribosomal RNA gene unification that serves to preserve the essential house-keeping functions of the converted genes. Transposed elements (TE), especially *Alu* short interspersed elements (SINE) that have more than a million copies in primate genomes, are a significant source of homologous units and a verified target of gene conversion. The consequences of such a recombination-based process are diverse, including multiplications of functional TE internal binding domains and, for evolutionists, confusing divergent annotations of orthologous transposable elements in related species. We systematically extracted and compared 68,097 *Alu* insertions in various primates looking for potential events of TE gene conversion and discovered 98 clear cases of *Alu*–*Alu* gene conversion, including 64 cases for which the direction of conversion was identified (e.g., *Alu*S conversion to *Alu*Y). Gene conversion also does not necessarily affect the entire homologous sequence, and we detected 69 cases of partial gene conversion that resulted in virtual hybrids of two elements. Phylogenetic screening of gene-converted *Alu*s revealed three clear hotspots of the process in the ancestors of Catarrhini, Hominoidea, and gibbons. In general, our systematic screening of orthologous primate loci for gene-converted TEs provides a new strategy and view of a post-integrative process that changes the identities of such elements.

## 1. Introduction

Genomes of most eukaryotic organisms contain a large number of repetitive sequences, a notable portion of which is composed of transposable elements (TE). For example, TEs occupy up to 69% of human genomes [1]. Despite the large numbers of TEs, only a few “master-copies” can actively propagate [2,3]. Accumulating changes in master copies leads to new subfamilies and types of TEs that commonly differ by several diagnostic sites and spread in limiting activity waves through the genome [4].

Due to their repetitive nature, high similarity, and large quantities in the genome, TEs present a significant substrate for non-allelic gene conversion. Gene conversion is a process whereby the genetic material of a donor sequence unidirectionally replaces that of a homologous acceptor sequence via recombination after a double-strand DNA break. Thus, gene conversion can proliferate mutations among TEs independently of the activity of the master-copy, leading to TE homogenization, a phenomenon known as concerted evolution [5]. Earlier retrotransposon studies reported a few cases of gene conversion between TE copies. For example, Kass and colleagues [6] described a case of gene conversion that changed a younger human *Alu* SINE to an older element. Roy et al. [7] suggested that gene conversion is responsible for ~10–20% of the variation in the young *Alu*Ya5 subfamily. A whole-genome gene conversion analysis among *Alu*s in humans [8] focused on non-diagnostic mutations in *Alu* sequences revealed significant levels of gene conversion, especially among neighboring *Alu*s. The authors found that gene conversion acts on *Alu*s within a range of about 10 kb, inversely proportional to their distance from one another. Most studies of gene conversion between TEs focused on *Alu* SINEs in primates. However, similar effects were also reported for LTRs in other mammals [9,10] and in plants [11].

As the vast majority of genomic TEs are neutral to the effects of natural selection, gene conversion does not usually have a crucial impact on the organism. However, TE gene conversion can have an adaptive effect in rewiring regulatory networks (reviewed in [12]). For example, gene conversion among ISX TEs might be responsible for optimizing binding sites for the dosage compensation complex in *Drosophila* [13].

Gene conversion may also directly influence the evolution of TEs [12,14]. Transferring mutations to master copies may increase or reduce their activity, as proposed for example for the *Alu*Yh3a3 subfamily [15]. Moreover, gene conversion might lead to the formation of new TE families or help maintain them in endosymbiont genomes by preventing their degradation and loss [16].

**Figure 1:**
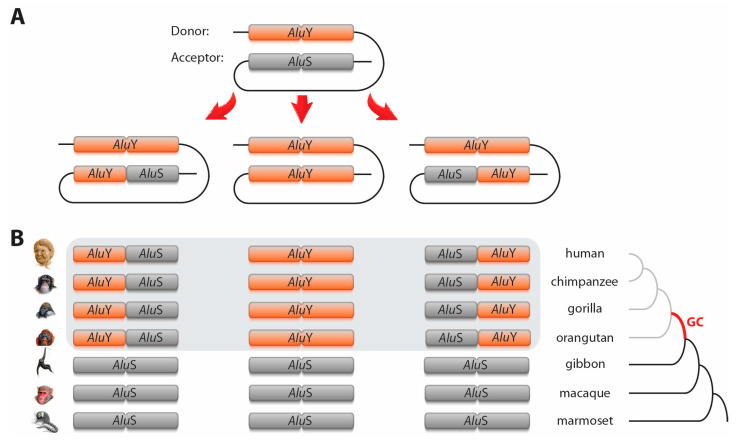
Schematic representation of *Alu*–*Alu* gene conversion. (**A**) shows three different scenarios of gene conversion: in which a 5′-end *Alu* monomer, a complete *Alu*, or a 3′-end *Alu* monomer were converted. (**B**) Representative gene conversion at orthologous *Alu*S loci leading to 5′-end *Alu*Y, *Alu*Y, and 3′-end *Alu*Y in the common ancestor of great apes (grey area; e.g., loci *Alu*S_plus_7691_7692, HGib37,f and H_*Alu*S6 in Supplementary Table S1 and Supplementary File S2, ignoring some variation in marmoset). The phylogenetic time-point of gene conversions is labelled GC (red branch of the tree).

The extent of sequence similarity between donor and acceptor loci positively influences the frequency of gene conversion, and reaches an optimum at 89%–100% [17,18]. Therefore, a substantial number of gene conversion events involves young TEs of the same subfamily. *Alu* elements are the most abundant TEs in primate genomes and have served as a model group for TE-based gene conversion studies e.g., [7,8]. *Alu*s evolved from 7 SL RNA around 65 million years ago in the ancestral lineage of primates and consist of dimeric sequences of about 300 nt (merged 5′- and 3′-monomers [19]). They diverged into three subfamilies/types—the oldest *Alu*J, the *Alu*S, and the youngest *Alu*Y. More than a million *Alu* copies are distributed across the human genome, occupying about 11% of genomic space [20]. Because gene conversion also acts on relatively short sequences (beginning with 10 nt [17]), not only are entire *Alu* sequences substituted, but also partial *Alu*–*Alu* gene conversion occurs, resulting in hybrid elements (e.g., hybrids with 5′-*Alu*S and 3′-*Alu*Y [21,22], Figure 1).

Changing the TE type via gene conversion might impact the global genome architecture and, for genome scientists, may also lead to faulty genome annotations and obstruct TE-based phylogenetic reconstructions. The phylogenetic presence of identical orthologous TE elements in several species indicates their close relationships, the identification of which can be compromised if gene conversion results in altered element types. We previously showed that parallel insertions and precise deletions of *Alu*s are rare in primates, confirming their usefulness as virtually homoplasy-free markers in phylogenetic studies [23]. However, no study has yet evaluated gene conversion as an additional possible source of confounding TE presence/absence patterns. Replacing one *Alu* type with another in a monophyletic species group can lead to an incorrect conclusion about their phylogenomic relationship. Therefore, to determine the extent of possible homoplasy caused by gene conversion and the frequency of gene conversion in TEs of different ages, we performed a systematic screening for gene conversion among *Alu* elements belonging to clearly different primate *Alu* subfamilies and types (*Alu*Y/*Alu*S and *Alu*Y/*Alu*Yc).

## 2. Materials and Methods

To identify potential incidences of *Alu* SINE gene conversion, we initially surveyed a published human genome RepeatMasker file to find all *Alu* SINE insertions. Because recurrent *Alu* clusters inhibit finding orthologous loci, we used fastCOEX to extract (nearly) complete human *Alu* elements with flanks largely free of additional TE sequences. The selected loci were then submitted to the 2-n-way computer suite to find all orthologous sequences of related primates. A subsequent RepeatMasker analysis of all individual hominoid TE regions revealed potential gene converted cases.

### 2.1. Hg38 Targeting AluY/Yc and AluS Sequences

We extracted target *Alu* element genomic coordinates from the human genome RepeatMasker outfile (December 2013, GRCh38/hg38) from the Genome Browser, University of California Santa Cruz (UCSC) (https://hgdownload.soe.ucsc.edu/goldenPath/hg38/bigZips/, (hg38.fa.out.gz), accessed on 9 June 2021). Using the fastCOEX program (http://retrogenomics.uni-muenster.de/tools/fastCOEX, accessed on 9 June 2021) [24], we selected coordinates of complete and nearly complete *Alu* elements (≤20 nt 5′-/3′-truncations) located in regions largely free from additional repeats (≥70% of 700 nt flanks repeat-free). Due to the high divergence of older *Alu*J elements from their consensus sequences, we restricted our analyses to younger *Alu*Y/Yc and *Alu*S elements.

### 2.2. Screening for Orthologous AluS/Y and AluY/Yc in Hominoidea

To screen for orthologous *Alu* elements in primates we used the 2-n-way suite [25]. We first generated the following 2-way whole-genome alignments (lastz algorithm): human/chimpanzee (hg38/Clint_PTRv2), human/gorilla (hg38/gorGor4), human/orangutan (hg38/Susie_PABv2), and human/gibbon (hg38/Nleu3.0). The 2-ways were then uploaded to the n-way module to perform human hg38 coordinate-based screenings for full-length *Alu*S, *Alu*Y, and *Alu*Yc elements and orthologous regions in other hominoids. N-way generated presence/absence tables, indicating the presence or absence of *Alu*s for all investigated primate species. We extracted sequences of loci in which (1) the targeted elements were present in all Hominoidea; (2) the targeted elements were present in all Hominidae except gibbon; and (3) the elements were present in human, chimpanzee, and gorilla but absent in orangutan and gibbon.

### 2.3. Detecting and Processing Cases of Alu Gene Conversion

Using a local version of the RepeatMasker (https://www.repeatmasker.org/RepeatMasker, accessed on 9 June 2021), we identified the family affiliations of *Alu*S, *Alu*Y, and *Alu*Yc hg38 orthologs for all investigated non-human primate species. For orthologous primate loci in which different *Alu* element subfamilies/types were detected (e.g., *Alu*Y in human vs. *Alu*S in chimpanzee), we performed an extensive alignment analysis using the Phylogenetic Data Editor (PhyDE, version 0.9971, Muenster, Germany). To help reconstruct the evolution of gene-converted loci we supplemented the analyses with orthologous sequences of bonobo and a second gorilla individual (gorGor5). To identify the ancestral states of the *Alu* insertions, we also analyzed the sequences of at least one representative Old World (*Macaca mulatta*) and New World (*Callithrix jacchus*) monkey outgroup. The macaque and marmoset sequences were retrieved via genome blast for potentially gene converted loci and added to the alignments. All alignments were supplemented with respective *Alu* consensus sequences.

We accepted specific *Alu* loci as gene conversion cases if orthologous elements were assigned to different element subfamilies/types based on at least three coherent diagnostic nucleotides for *Alu*S/Y or diagnostic indels for *Alu*Y/Yc (Supplementary File S1). For *Alu*S/Y gene conversion, we accepted cases where the *Alu*s belonged to one of the *Alu*S and *Alu*Y subfamilies (including *Alu*Yc), respectively. For *Alu*Y/Yc gene conversion, we considered all *Alu*Ys (without 12-nt diagnostic deletion at the 5′-end of the consensus) and all *Alu*Ys with the specific deletion (*Alu*Yc, Yc3, Yc5, Yd2, Yd3, Yd8), respectively. Furthermore, we accepted as gene converted only those loci with deviating *Alu* types from species that were phylogenetically nested within their related primates (Figure 1). These decisions were based on the concepts that orthologous *Alu*s of differing element subfamilies and types in various related primates cannot be explained solely by single events of independent insertions and must instead be attributed to gene conversion. For example, to explain the presence of an *Alu*Y in humans and an *Alu*S in all other hominoids by orthologous independent insertions or precise deletions, we would require five independent insertions (*Alu*Y in the human lineage and four independent insertions of *Alu*S in the different hominoid lineages) or, alternatively, an *Alu*S insertion in the ancestral lineage of Hominoidea with a subsequent precise deletion and precise insertion of *Alu*Y in the human lineage. Another conceivable scenario includes the specific accumulation of mutations in the diagnostic positions leading by chance to a change of *Alu* types. However, we accepted only cases in which at least three specific neighboring mutations were necessary to change the *Alu* subfamily or type. Thus, for all these cases, a single gene conversion event represents the most parsimonious explanation.

### 2.4. Screening for Hybrid Elements in Human

To search for hybrid *Alu*s in the human genome, we conducted additional analyses. *Alu* elements were extracted as described in Section 2.1 and the sequences then split into 5′- and 3′-monomers. We ran a local repeat masking for both datasets independently and compared the *Alu* subfamily/type affiliations of 5′- and 3′-monomer pairs. If they were assigned to different subfamilies/types by at least three coherent diagnostic changes, we performed a manual analysis including other primate species (see Section 2.3) and reconstructed the evolutionary history of gene conversion.

### 2.5. Screening for Potential Polymorphic Gene Conversion in the Human Population

We supplemented the loci with gene conversion in the human lineage (after the human-chimpanzee split) with genomic information of additional human individuals. We retrieved 35 human genomes available in NCBI for which the ethnical origin was described.

### 2.6. Counting of Gene Converted Alu TEs

We applied two quality controls: (1) at least two sources of data (assemblies, individuals, species) were used to verify gene conversion, (2) restricting comparisons to secured orthologs by omitting “low complexity” concatenated *Alu* element regions. To evaluate the “lowest verifiable level” of homoplasy caused by *Alu*–*Alu* gene conversion, we followed the calculation published in Doronina et al. for homoplasious deletions and insertions [23]. We applied the human-chimpanzee-rhesus macaque model group and focused on *Alu*s inserted before the Catarrhini diversification. Accordingly, we calculated the ratio of gene converted *Alu*s in the human and chimpanzee lineages compared to the total number of *Alu* insertions in the common ancestor of Catarrhini.

## 3. Results and Discussion

Here we present for the first time a systematic, genome-wide screening of primate genomes for clear *Alu*–*Alu* element type change via gene conversions. Two recently developed tools were combined to find 98 specific cases of gene conversion. fastCOEX derived *Alu* loci with almost TE-free flanks, and 2-n-way extracted their orthologous sequences in various primate species. Gene conversion is identifiable when different *Alu* subfamilies or types recombine (e.g., *Alu*S change to *Alu*Y or vice versa). From a RepeatMasker report of the human genome using fastCOEX [24], we extracted human coordinates of 55,408 *Alu*S and 12,689 *Alu*Y/Yc full-length elements with flanking regions largely free of other repetitive sequences. However, restricting our screening to these most reliable cases of *Alu* TEs reduces the total dataset of human *Alu*s (~800.000 for *Alu*S plus *Alu*Y) by about a tenth. We used the 2-n-way computer suite to retrieve 46,285 targeted *Alu*S and 8099 *Alu*Y/Yc elements orthologous loci for a set of hominoid species (see Section 2.2 under Methods). We then applied a local RepeatMasker analysis to both annotate each hominoid insertion at orthologous positions, and, in a search for human hybrid elements, to compare the element subtypes of 5′- and 3′-*Alu* monomers for each human *Alu*. After manual inspection to verify orthology, we identified 98 cases in which some primate species or species groups contained different *Alu* elements or hybrid *Alu*s compared to the others in the group (Table 1, Supplementary Table S1, and Supplementary File S2). It has to be mentioned that this number underestimates the actual extent of *Alu* gene converted loci. The more similar elements are, the more probable they involve in gene conversion. However, gene conversion of identical elements is difficult to trace. About half of the identified gene converted *Alu* elements were located in gene regions (introns or UTRs). The other half was found in the intergenic areas of the genome (Supplementary Table S1). However, because of restricting our survey to *Alu*s free from flanking TEs, we underestimate the portion of *Alu*–*Alu* gene conversion in intergenic regions.

For 64 of the 98 gene conversion loci, we were able to reconstruct the original ancestral *Alu* element type and to determine the direction of gene conversion. For the *Alu*S to *Alu*Y conversions (40 loci; Table 1, first three lines), the older *Alu*S elements (*Alu*Ss ceased their main activity before the diversification of Catarrhini) were replaced by younger, potentially active *Alu*Y elements (*Alu*Ys exhibited their main activity starting with the divergence of Catarrhini) [26]. This suggested that young, actively transcribed DNA regions were the preferred donors for gene conversion [27]. However, we also observed incidences in which the reverse process occurred (12 cases; Table 1, line 4–6), providing evidence that old inactive elements might replace young active elements via gene conversion resulting in a sort of “life after death” spreading throughout the genome after silencing. For 14 of the reconstructed 52 loci involving both *Alu*S and *Alu*Y elements, we detected gene conversion of the complete acceptor element, whereas in the remaining 38 loci only partial gene conversion occurred, leading to “mosaic” or hybrid elements (e.g., a hybrid of *Alu*S 5′-monomer and *Alu*Y 3′-monomer). It should be mentioned that *Alu*Y/*Alu*S gene conversion events resulted in hybrids of *Alu*Y 5′-monomer and *Alu*S 3′-monomer (12 cases) can also be potentially *Alu*Sc8/*Alu*S gene conversion because the 5′-monomer of *Alu*Sc8 shares the diagnostic mutations of *Alu*Y and the 3′-monomer of *Alu*S. Furthermore, we detected an additional 31 cases of hybrid elements, in which we were unable to assign the pre-conversional state of the *Alu* elements (Table 1, line 9). We were unable to categorize *Alu*Y/*Alu*S hybrids for cases of unidentified ancestral origins because they were indistinguishable from *Alu*Sc8 elements. We also observed 12 incidences of gene conversion among *Alu*Y and *Alu*Yc elements and 3 cases, in which the *Alu* loci underwent more than one gene conversion event during primate evolution (Table 1).

**Figure 2:**
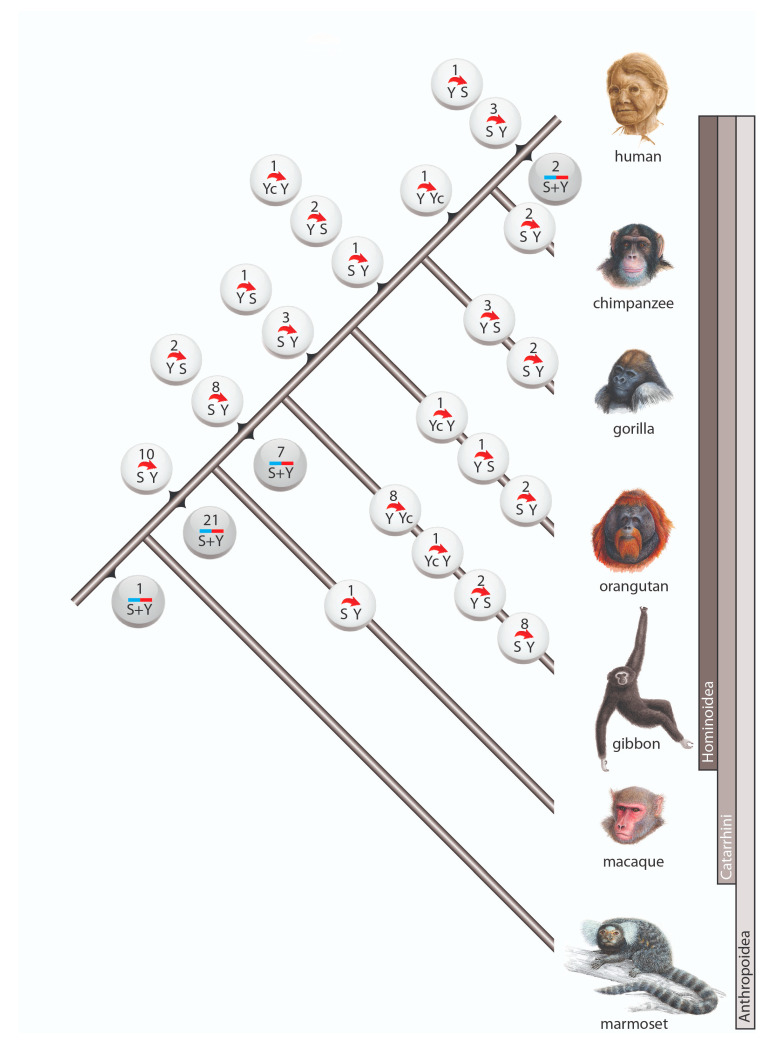
Gene conversion in primates. Circles represent incidences of gene conversion including the number of such occurrences and their direction. White circles are incidences in which the ancestral *Alu* element and the direction of conversion were reconstructed. Gray circles represent incidences with unidentifiable conversion direction. The 3 cases with complex scenarios are not shown.

Among the 98 cases of gene conversion, 64 occurred on the lineage leading to humans (including 6 instances after human split from chimpanzee), whereas 31 gene conversions occurred on the terminal branches of other investigated primates (Figure 2). Within Anthropoidea we distinguished three waves of high gene conversion events: (1) on the ancestral branch of Catarrhini (31 conversions), (2) on the ancestral branch of hominoids (17 conversions), and (3) in the gibbon lineage (19 conversions). The first two of these higher incidences might be explained by the longer lengths of the ancestral internodes leading to Catarrhini and hominoids, both leaving substantial times for the occurrence and fixation of gene conversion events. The increased gene conversion events in gibbons might be partially explained by the more highly active gibbon-specific *Alu*Y elements (*Alu*Yd3a1_gib [28]), which contain the same diagnostic deletion as the *Alu*Yc element.

Another gene conversion-rich branch was that leading to gorilla. In our initial analysis, we screened the gorGor4 genome assembly (gorilla Kamilah, UCSC https://genome-euro.ucsc.edu/cgi-bin/hgGateway, accessed on 9 June 2021) and found 12 gene conversions (Supplementary File S3). A previous examination of interlocus gene conversion in gorGor4 [29], also observed a more frequent occurrence of gene conversion in gorilla than in other great apes. However, our expanded analysis of another gorilla genome (gorilla Susie, gorGor5) revealed only 5 gene conversion events (Figure 2, Supplementary Table S1), suggesting that the difference between gorGor4 and gorGor5 is an individual variation or a genomic artifact of the gorGor4 assembly. The gorGor6 assembly (August 2019, assembly Kamilah_GGO_v0/gorGor6) that recently became available carries none of the previously detected cases of gene conversions found solely in gorGor4, suggesting there might be assembly errors in gorGor4. Learning from gorilla, we compared gene conversion patterns for at least two related species or independent assemblies in cases when gene conversion occurred on a terminal branch to avoid such assembly issues.

We conducted a population analysis of human-specific gene conversions (6 cases), including 35 human individual genomes from Africa, Asia, America, and Europe (Supplementary Table S2). We found a consistent gene conversion pattern in all investigated genomes for the 5 loci containing *Alu*S to *Alu*Y conversions. For the remaining one locus (*Alu*Y converted to *Alu*S), gene conversion was only detected in some human individuals. Contrary to our expectations, we could not find a phylogenetic pattern of the gene conversion distribution among 35 individuals. Orthologous *Alu* gene conversion was found in 2 of 9 African individuals, 5 of 13 Asian, 1 of 4 American, 1 of 1 Puerto Rican, and 5 of 8 European individuals. We suggest that such a mosaic of gene conversion events might result from duplication of *Alu* loci in the human genome with the subsequent gene conversion in one of the copies. Alternatively, multiple independent conversions could have occurred.

In the present study, we examined gene conversion events leading to changes in the *Alu* subfamily or type affiliations in selected hominoids. It should be noted that because of sequence similarity, gene conversion occurs most frequently among identical or closely related elements, and is then unrecognizable. Here we showed that *Alu*S/Y/Yc gene conversion occurred in all hominoid lineages. We suggest that the observed patterns of *Alu*–*Alu* gene conversion in hominoids are also representative of other primate species and TE types.

Parallel insertion, exact deletion, or gene conversion might lead to apparently conflicting presence/absence patterns at orthologous loci. Doronina et al. [23] showed there to be a negligibly low frequency of conflicting phylogenetic signals amongst *Alu* elements in primates. However, they did not examine gene conversion. Although Aleshin et al. [8] found a notable quantity of potential *Alu*–*Alu* gene conversions, their screening method (ignoring diagnostic *Alu* positions) does not evaluate the contribution of gene conversion to homoplasy. Similar to the data in Doronina et al. [23], we estimate the frequency of gene conversion-related homoplasy in the human-chimpanzee-rhesus macaque model group to be 0.0006% in human (3/544,034 × 100%) and 0.0004% in chimpanzee (2/544,034 × 100%), where 544,034 is the number of *Alu* insertions present in the Catarrhini ancestral lineage. Thus, we provide evidence for the existence of homoplasy caused by gene conversion, but show that the frequency is even lower than parallel insertions or precise deletions.

It should be mentioned that the classical, distance-based (the divergence of a TE sequence from a consensus sequence) calculations of the ages of TEs used in evolutionary studies might be distorted by gene conversion [9,11,30]. Our results suggest that transposition-in-transposition-based analyses [26] that take into account element types rather than accumulated mutations in TE sequences may provide a more reliable alternative. Indeed, we detected relatively few gene conversion events per lineage affecting diagnostic positions that resulted in TE subfamily or type changes, whereas the sharing of non-diagnostic mutations among *Alu*s via gene conversion was shown to be a frequent phenomenon [8].

In summary, the footprints of gene conversion are directly detectable by genome-wide comparisons of deviating annotations of orthologous TEs in different species (e.g., orthologous *Alu* SINEs with different subfamily or type affiliations in primates). Many potential incidences of partial gene conversion were detected that resulted in hybrid elements. Incidences of gene conversion in TEs are frequent enough to visualize by genome-level screenings but rare enough that they do not challenge large-scale phylogenetic TE presence/absence studies.

## Figures and Tables

**Table 1:** Gene conversion cases among *Alu* elements.

Donor	Acceptor	Replaced Part of *Alu*	Number of Loci
Gene conversion with identified direction			
*Alu*Y	*Alu*S	Complete *Alu*	11
*Alu*Y	*Alu*S	3′-*Alu* unit (S-Y hybrid)	23
*Alu*Y	*Alu*S	5′-*Alu* unit (Y-S hybrid)	6
*Alu*S	*Alu*Y	Complete *Alu*	3
*Alu*S	*Alu*Y	3′-*Alu* unit (Y-S hybrid)	6
*Alu*S	*Alu*Y	5′-*Alu* unit (S-Y hybrid)	3
*Alu*Yc	*Alu*Y	Diagnostic indel	9
*Alu*Y	*Alu*Yc	Diagnostic indel	3
Gene conversion with unidentified direction			
Unidentified	Unidentified	*Alu*S–*Alu*Y hybrid	31
Complex scenario	Complex scenario	Multiple gene conversion	3

## Data Availability

All data are presented in Supplementary Files.

## Supplementary Materials

The following are available online at https://www.mdpi.com/article/10.3390/genes12060905/s1, Table S1: List of 98 *Alu*–*Alu* gene conversion loci and their genomic and species context, Table S2: Information of 35 additional human individuals including in the analyses for the six human-specific *Alu*–*Alu* gene conversion, Supplementary File S1: Pairwise alignment of subfamily or type *Alu*Y/*Alu*Sz and *Alu*Y/*Alu*Yc sequences, Supplementary File S2: Alignments of 98 *Alu*–*Alu* gene conversion loci. Supplementary File S3: Coordinates of *Alu* elements in human, which were identified as cases of *Alu*–*Alu* gene conversion based on gorilla assembly gorGor4.
